# Experiences of visiting female sex workers, social interaction, support and HIV infection among elderly men from rural China

**DOI:** 10.1186/s12879-023-08178-z

**Published:** 2023-05-25

**Authors:** Yi Yang, Shu Liang, ShuangFeng Fan, Yang Liu, Yuan Li, Jing Xi, Dan Yuan, Jie Xiao

**Affiliations:** 1 School of Management, Chengdu University of Traditional Chinese Medicine, 1166 Liutai Avenue, Wenjiang District, Chengdu, 611137 China; 2 Institute of HIV/AIDS prevention, Sichuan Center for Disease Control and Prevention, No.6, Zhongxue Road, Wuhou District, Chengdu, 610000 China; 3 Department of HIV/AIDS prevention, Chengdu Center for Disease Control and Prevention, No.4, Longxiang Road, Wuhou District, Chengdu, 610000 China; 4 School of Public Health, Chengdu University of Traditional Chinese Medicine, 1166 Liutai Avenue, Wenjiang District, Chengdu, 611137 China

**Keywords:** Social Support, HIV, Elderly men, Client of female sex workers, Case-control study

## Abstract

**Objectives:**

To make clear the roles of social interaction and social support in HIV infection among elderly men who visit female sex workers (FSW).

**Methods:**

We conducted a case-control study: 106 newly HIV (+) vs. 87 HIV (-) elderly men who visited FSW with similar age, education levels, marital statuses, monthly expenses for entertainment and migration experiences. Experiences of visiting FSW, social interaction, and intimate social support were obtained. Backward binary logistic regression was applied.

**Results:**

Cases’ first visit to FSW happened at the age of 44.01 ± 12.25, older than controls (33.90 ± 13.43). 23.58% cases had gotten HIV-related health education (HRHE) before, less than controls (57.47%). More cases (48.91%) “always” got material support than controls (34.25%). Less cases gave “close” (38.04%) comments toward daily life, “satisfied” (34.78%) with their sexual life, “agree” being emotional fulfilled (46.74%) than controls (71.23% ,64.38%, and 61.64%). Risky factors for HIV infection among elderly men were having 3000 YUAN and above monthly income, visiting teahouse with friends, living without spouses, visiting different FSW, visiting FSW for other reason, receiving material support from most intimate sexual partner, older age of first visit to FSW. The protective factors were receiving HRHE, visiting FSW due to loneliness, and giving positive comments toward daily life with most intimate sexual partner.

**Conclusions:**

Elderly men’s social interactions are mainly visiting teahouse which is a potential sexual venue. Getting HRHE is formal protective social interactions but very rare for cases (23.58%). Social support from sexual partner is not enough. Emotional support is protective meanwhile material support only is risky for becoming HIV-positive.

## Introduction

Similar as in the world [1], China is facing big challenges to achieve “90–90–90 targets”, namely 90% of people living with HIV (PLHIV) knowing their status, 90% of those diagnosed receiving sustained ART, and 90% of those receiving ART achieving viral suppression by 2020 and to end the HIV/AIDS epidemic as a public health threat by 2030 [2]. One of the biggest challenges is that with population aging [3], HIV prevalence of elderly(≥ 50 years old [4–6]) men in China has been steadily increasing mainly through heterosexual transmission [5, 7, 8], much higher than the general population [9]. Due to immune functional decline and increased vulnerability to infections [10], multiple morbidity [11], facing stigma [12], HIV infected elderly men quickly progress into AIDS even deaths [7, 10]. Before they are confirmed as HIV-positive, with low condom use [13, 14], they act as route of HIV transmission unintentionally. In 2019 one year “90–90–90 targets” supposed to be achieved, we conducted a survey from rural China and found out that 21.51% elderly men never heard about HIV/AIDS.

Though there are 190.59 million population age 65 + in China in 2020 (from 6.96% to 2000 to 13.50%) [15], HIV/AIDS prevention programs focusing on elderly men have just started. Elderly men who are considered as sexually inactive [16] had been excluded from the priority of HIV/AIDS prevention programs [17–20], and were not listed as a key prevention group until 2017 in China [4]. Traditionally, China is a patriarchy society dominantly influenced by Confucism [21, 22]. Sex has been taken as procreation within a family context. People don’t talk about sex [23, 24]. Since the 1980s, when migrating alone, due to lack of norms and values’ control from original communities [13, 23, 25–30], sexual attitudes in China have changed to being an individual’s responsibility as long as there is no foreseen negative impact on the well-being of others or the larger society [23]. Current elderly men were easy to be involved in high-risk sexual behaviors such as visiting female sex workers (FSW) and having sex with men when they were sexually active 20-50-year-old and migrated [13, 23, 25–30]. Compared with other age groups, elderly men have minimal knowledge about HIV/AIDS [31], condom use among them is lower than younger population [13, 14].

Visiting FSW has been taken as the major cause of HIV epidemic among elderly men in China [5, 7, 8], not just to fulfil physical need but also emotional need [13, 23, 25–30]. The closer clients feel with FSW, the less they use condom consistently [13]. Feeling lonely rather than social isolation is risky for mental health problems [32, 33]. Social isolation as the result of reduced social interactions due to retirement, shrinking network size, living alone and so on is more prevalent among elderly population than general population, is threating their mental and physical health [34, 35], and need more studies to confirm the mechanisms [36].

Social support which helps to maintain well-being [37] for elderly men in China is insufficient. Traditionally, men are supposed to be a “giver” rather than a “receiver” [21, 22]. Elderly population with greater social support are more likely to do physical activities at their leisure time [38], and reduce depression (around 13.3% globally) [39]. Low social support is found out being associated with HIV-related stigma, which brings in detrimental impact on a variety of health-related outcomes [40, 41].

Therefore, we would like to know why some elderly men who visit FSW get HIV infected, some not? What are the roles of social interaction and social support in HIV infection among elderly men who visit female sex workers (FSW)?

## Methods

### Study site

Chengdu, the capital city of Sichuan province, is one of the suitable study sites in China. One of fifth people living with HIV/AIDS (PLHIV) in Sichuan are from Chengdu. Located in southwest of China, Sichuan province is one of the few provinces in China heavily affected by the HIV/AIDS epidemic, though successful comprehensive HIV prevention strategies has been applied [2]. Elderly people in Sichuan accounted for less than 5% of HIV/AIDS cases in 2005, and over 40% in 2017, and 59.2% in 2019 [2, 5], meanwhile the proportion of elderly men in the population is less than 40% [42] .

With the coverage of sentinel surveillance improving [2] and around 800,000 population [43], rural County A in Chengdu ranked one of the top five counties in the number of PLHIV, mainly from male group [43].

### Study design and Participants

We conducted a case-control study comparing 106 newly diagnosed HIV-infected elderly men with 87 HIV-uninfected elderly men. Eligibility criteria for case group included: (1) ≥ 50 years old; (2) male; (3) live in the current address for at least six months;(4) newly diagnosed HIV (+); (5) self-report visiting FSW in their lifetime; (6) willing to participate the study, and sign informed content. We also enrolled controls, those who were HIV (-). Exclusion criteria for both groups included: (1)cannot speak clearly; (2) with mental problems and recall disability [42, 44–46].

Based on China National Guideline for Detection of HIV/AIDS, Dot immunocolloid gold rapid test (Yingke New Technology Co., Ltd.) or enzyme-linked immunosorbent assay (ELISA) was used for HIV antibody test as HIV rapid testing. When HIV (+) results were obtained from HIV rapid testing, 3–5 ml cubital venous blood was sampled and sent to local HIV confirmation laboratory. Immunoblotting (WB) was applied for HIV antibody confirmatory test. During April 2019 and October 2020, 115 elderly men from 8 townships in County A were confirmed as HIV (+), and 114 participated the study. 106 of 114 (92.98%) reported visiting FSW in their lifetime, and were included in this study as cases.

During June to July 2019, a cross-sectional study from the same townships was conducted, 802 men were recruited, and 797 (99.38%) questionnaires were reliable, and among them 795 were HIV (-) [6]. 88 of 795 (11.07%) HIV (-) respondents admitted visiting FSW in their lifetime, and 87 of 88 HIV (-) respondents provided detailed information about visiting FSW and were included in this study as controls.

## Data Collection

A written informed consent was completed before the investigation. Local slangs were used to refer sexual behaviors. A structured questionnaire was applied for both cases and controls. In-depth interviews with one case were conducted by one skilled staff from County A center for disease control and prevention (CDC) within one month after cases’ HIV diagnosis. In total, two medical staff conducted the surveys with all 106 cases in separate rooms in CDC, county infectious hospital, or township health centers based on cases’ convenience. A 30-minute face-to-face structured interview with a control was conducted in a separate room at the village health centers to make sure the respondents felt comfortable to talk about their sexual behaviors by one well-trained male interviewer.

## Measures

### Basic information

demographic characteristics, migration experiences, sexual desire change after the age of 50, and attitude toward visiting FSW.

### Experiences of visiting female sex workers

Time, site, channels of knowing sexual venues, and reasons for their first visit to FSW, visiting preferences in their lifetimes. Number of their sexual partners, condom use with FSW and reasons for no condom use in the past three years.

Optional reasons for their first visit to FSW in the past three years are: (1) “dissatisfied with marital sexual life”; (2) “divorced/single, purely fulfil sexual desire”; (3) “accompany with friends/colleagues” ;(4) “lonely”; (5) “under bad mood”; (6) “seduced by FSW”; and (7) “other reasons”.

### Social interaction

Social interaction was measured into two categories: **(1) common life interaction–** types of daily entertainment, including: visiting teahouse with friends, playing mahjong, tourism, parties, exercises, square dancing, watching TV and other; **(2) HIV-related health education (HRHE)** as asking “whether receiving HRHE before the interview or before the HIV (+) confirmation or not.” For the one who received HRHE, optional channels were asked including: ①health workers from CDC; ②health workers from township health centers and village doctors; ③doctors from other kind of hospitals; ④children/grandchildren’s teachers;⑤civil servant from township government; ⑥village cadres; ⑦pharmacy staff; ⑧volunteers; ⑨other informal channels, such as family members, sexual partners, owners of sexual venues, and so on.

### Intimate social support

Intimate social support from their sexual partners within the past three years was measured: (1) **material support**: “If you were lack of money, would he/she provide economic/material support as you needed?” The response options were: *never, occasionally, often, always*. (2) **emotional support**: “she/he bring in emotional fulfillment to you” with response options as *strongly disagree, disagree, undecided, agree, strongly agree*. Comments toward sexual life with response options as *extremely dissatisfied, dissatisfied, undecided, satisfied, extremely satisfied.* Comments toward daily life with response options as *extremely alienated, alienated, undecided, close, extremely close.*

Frequencies for nominal variables, mean and standard deviation for interval variables were assessed. T test and Chi-square tests/ fisher’s exact test were used to examine the relationships between HIV infection and independent variables. Binary logistic regression with backward selection was applied to examine factors associated with HIV infection with *P < 0.05* in bivariate analyses. Adjusted odds ratio (AOR) and 95% confidence intervals were calculated. Factors with AOR greater than one was categorized as risk factors, and less than one as protective factors.

## Results

### Basic information

The age was 62.88 ± 8.50 for cases, 61.57 ± 7.11 for controls, the difference was not statistically significant (t = 1.14, P = 0.26), similar with the differences of education levels, marital statuses, monthly expenses for entertainment and migration experiences between two groups*(P > 0.05*). In terms of types of residencies, latest occupations, types of living with, monthly income, the differences between two groups were statistically significant*(P < 0.05)*, details are showed in Table 1.

**Table 1:** Basic Characteristics Comparison Between HIV (+) vs. HIV (-) Elderly men in Chengdu, China (n = 193, n (%))

	HIV (+) (n = 106)	HIV (-) (n = 87)	χ^2^	*P*
**Type of residency**			4.13	0.04
City	12(11.32)	3(3.45)		
Rural	94(88.68)	84(96.55)		
**Education level**			2.93	0.71
Illiterate	16(15.09)	15(17.24)		
Primary school drop outs	37(34.91)	25(28.74)		
Primary school	15(14.15)	14(16.09)		
Junior high school drop outs	15(14.15)	9(10.34)		
Junior high school	19(17.92)	22(25.29)		
Senior high school/technical school	4(3.77)	2(2.3)		
**Marital status**			1.31	0.52
Married	71(66.98)	62(71.26)		
Never married	5(4.72)	6(6.9)		
Single(divorce/widow/separated)	30(28.3)	19(21.84)		
**Latest occupation**				< 0.01*
Farm workers/workers	95(89.62)	62(71.26)		
Government employees	9(8.49)	19(21.84)		
Other	2(1.89)	6(6.9)		
**Living with**			9.38	0.03
Only spouse	25(23.58)	33(37.93)		
Spouses and other family members (parents or children)	36(33.96)	32(36.78)		
Only other family members (parents or children)	25(23.58)	8(9.2)		
Nobody	20(18.87)	14(16.09)		
**Monthly income**			17.62	< 0.01
< 1000 YUAN	27(25.47)	43(49.43)		
1000–1999 YUAN	21(19.81)	21(24.14)		
2000–2999 YUAN	22(20.75)	11(12.64)		
3000 YUAN and above	36(33.96)	12(13.79)		
**Monthly expense for entertainment**				0.39^*^
< 500 YUAN	61(57.55)	53(60.92)		
500–999 YUAN	26(24.53)	27(31.03)		
1000–1499 YUAN	11(10.38)	4(4.6)		
1500–1999 YUAN	3(2.83)	1(1.15)		
2000 YUAN and above	5(4.72)	2(2.3)		
**Migration history**			0.30	0.86
More than one year ago	56(52.83)	48(55.17)		
Within the past one year	28(26.42)	20(22.99)		
Never	22(20.75)	19(21.84)		

*: fisher’s exact test

## Sexual desire change and attitude toward FSW

81 of 106 (76.42%) cases and 67 of 87 controls (77.01%) reported that their sexual desire has declined after the age of 50 (*P > 0.05*). 59 of 106 (55.66%) cases said commercial heterosexual behavior is understandable as natural human behavior, however 43.40% held undecided standpoint, and 0.94% reported that they could not accept. The corresponding responses among controls were 88.51%, 4.60% and 6.90%(*P < 0.01*).

## Experiences of visiting female sex workers

Cases visited FSW for the first time at the age of 44.01 ± 12.25, older than controls (33.90 ± 13.43, *P < 0.01*). Cases’ first FSW visit happened mainly within their township (59.43%), more than controls (42.53%, *P < 0.05*). Only 28.72% cases’ first FSW visit happened at migrate sites, much lower than among controls (83.82%, *P < 0.01*). Channels of knowing sexual venues were similar among cases and controls, mainly “following friends” (47.17% vs. 43.02%) and “heard from someone” (32.08% vs. 40.70%) (*P* > 0.05). More cases visited FSW due to “other reasons” (41.50%) and “dissatisfied with marital sexual life” (31.13%), “seduced by FSW” (27.35%) than controls (6.90%, 4.60%, and 8.05%, *P < 0.01*), and less due to “divorced/single, purely fulfil sexual desire” (29.24%) and “lonely” (22.64%) than controls (60.92% and 52.87%, *P < 0.01*). The reasons as “accompany with friends/colleagues” (44.34%) and “under bad mood” (8.49%) among cases were similar with among controls (43.68% and 5.75%, *P > 0.05*). Cases preferred to visit different FSW each time (54.72%), by random (44.34%), and visited a few fixed FSW (0.94%), no one visited only one fixed FSW, different from controls (62.07%, 18.39%, 5.75% and 13.79%, *P < 0.01*).

In the past three years, 72 of 106 (67.92%) cases reported visiting FSW, compared with only 16 of 87 (18.39%) controls (*P < 0.01*). Among them, the percentages of “never” “occasionally” and “often” used condom were 90.28%, 9.72% and 0.00% among cases, 68.75%,12.50% and 18.75% among controls (*P < 0.01*). Major reason for no condom use during last visiting FSW was “feeling uncomfortable” among cases (54.17%) and “don’t how to use condom due to never use” (43.75%) among controls (*P < 0.01*).

## Social interaction

Cases visited teahouse with friends (86.79%) and watched TV (81.13%) more than controls (74.71% and 44.83%, *P < 0.05*). Only 25 of 106 (23.58%) cases had gotten HRHE before, lower than controls (50/87, 57.47%) (*P <* 0.05). Neither of cases nor controls had gotten HRHE from pharmacies, HIV-related service volunteers and their children/grandchildren’s teachers. Cases had gotten HRHE less from health workers from township health center/village doctors (8.00%), health workers from CDC (0.0%,), village cadres (4.00%), and more from others (80.00%) than controls (60.00%,36.00%, 14.00% and 8.00%) (P < 0.05). Details are showed in Table 2.

**Table 2:** Social Interaction Between HIV (+) vs. HIV (-) Elderly men in Chengdu, China (n = 193, n (%))

Social interaction		HIV (+) (n = 106)	HIV (-) (n = 87)	χ^2^	*P*
**Daily life interaction**	Visiting teahouse with friends	92(86.79)	65(74.71)	4.60	0.03
	Playing mahjong	60(56.6)	46(52.87)	0.27	0.60
	Tourism	6(5.66)	3(3.45)		0.52*
	Parties	18(16.98)	13(14.94)	0.15	0.70
	Exercises	5(4.72)	8(9.2)	1.53	0.22
	Square dancing	1(0.94)	0(0)		1.00*
	Watching TV	86(81.13)	39(44.83)	27.60	< 0.01
	Other	22(20.75)	12(13.79)	1.60	0.21
**Gotten HIV-related Health Education before**		25(23.58)	50( 57.47)	23.09	< 0.01
**HRHE Provider**		**HIV (+)** **(n = 25)**	**HIV (-)** **(n = 50)**		
	Health workers from CDC	0(0)	18(36.00)	23.09	< 0.01
	Health workers from township health center/village doctors	2(8.00)	30(60.00)	11.84	< 0.01
	Doctors from other hospitals	0(0)	2(4.00)		0.55*
	Township civil servants	0(0)	8(16.00)		0.046*
	Village cadres	1(4.00)	7(14.00)		0.26*
	Others	20(80.00)	4(8.00)	39.71	< 0.01

*: fisher’s exact test

## Social support from the most intimate sexual partner

Cases reported 29.40 ± 48.15 sexual partners during the past three years, much more than controls (1.91 ± 4.36, P < 0.01). More cases (48.91%) “always” got material support from their most intimate sexual partners than controls (34.25%). Less cases gave “close” (38.04%) comments toward daily life, “satisfied” (34.78%) with their sexual life, “agree” being emotional fulfilled (46.74%) than controls (71.23% ,64.38%, and 61.64%, P < 0.01). Details are showed in Table 3.

**Table 3:** Social support from most intimate relationship sexual partner(n = 165)

		HIV (+) (n = 92)	HIV (-) (n = 73)	χ^2^	*P*
If you were lack of money, would he/she provide economic/material support as you needed?	Never	39(42.39)	32(43.84)		0.02*
	Occasionally	4(4.35)	13(17.81)		
	Often	4(4.35)	3(4.11)		
	Always	45(48.91)	25(34.25)		
Comments toward daily life	Extremely alienated	19(20.65)	0(0)		< 0.01*
	Alienated	10(10.87)	1(1.37)		
	Undecided	24(26.09)	18(24.66)		
	Close	35(38.04)	52(71.23)		
	Extremely close	4(4.35)	2(2.74)		
Comments toward sexual life	Extremely dissatisfied	1(1.09)	0(0)		< 0.01*
	Dissatisfied	18(19.57)	3(4.11)		
	Undecided	41(44.57)	21(28.77)		
	Satisfied	32(34.78)	47(64.38)		
	Extremely satisfied	0(0)	2(2.74)		
She/he bring in emotional fulfillment to you	Strongly disagree	5(5.43)	0(0)		< 0.01*
	Disagree	15(16.3)	2(2.74)		
	Undecided	29(31.52)	20(27.4)		
	Agree	43(46.74)	45(61.64)		
	Strongly agree	0(0)	6(8.22)		

*: fisher’s exact test

## Multiple reasons for becoming HIV-positive

Risky factors for HIV infection among elderly men were having 3000 YUAN and above monthly income, visiting teahouse with friends, living without spouse, visiting different FSW, visiting FSW for other reason, receiving material support from most intimate sexual partner, older age of first visit to FSW. The protective factors were receiving HRHE, visiting FSW due to loneliness, and giving positive comments toward daily life with most intimate sexual partner. Details are showed in Table 4.

**Table 4:** Association between HIV infection and related factors among Elderly men in Chengdu, China, backward logistic regression (n = 165)

	aOR (95%CI)
**Monthly income (vs. <1000 YUAN)**	
1000–1999 YUAN	4.63(0.56–38.7)
2000–2999 YUAN	11.94(0.89-159.24)
3000 YUAN and above	257.6(13.07-5075.54)
**Visiting teahouse with friends (yes vs.no)**	44.95(2.95-684.62)
**Living with (vs. Only spouse)**	
Spouses and other family members	0.28(0.04–2.11)
Only other family members	13.55(1.16-158.89)
Nobody	19.82(1.43-274.34)
Preference for FSW visit (only one fixed FSW- by random, 1–4)	18.59(3.29-104.95)
Reason for FSW visit -other (yes vs. no)	17.29(1.23-242.82)
Material support (never to always, 1–4)	4.41(1.71–11.39)
Age of first visit to FSW (17–77, every one year)	1.07(1.004–1.13)
Receiving HIV-related Health Education (yes vs. no)	0.01(0.001–0.12)
Reason for FSW visit -loneliness (yes vs. no)	0.05(0.00-0.55)
Comments toward daily life (extremely alienated to extremely close, 1–5)	0.23(0.08–0.63)
Comments toward sexual life (extremely dissatisfied to extremely satisfied, 1–5)	0.34(0.11–1.11)

## Discussion

Social interaction and material support without enough emotions is risky for elderly men who visit FSW. In 2020, disposable per capita income of Sichuan residents is around 2200 Yuan [44], and visiting FSW in County A only cost around 20–49 YUAN each time [6]. 3000 YUAN monthly income become the threshold of visiting FSW without financial limitation in this study. The more frequently visit FSW, the more easily elderly men become HIV infected. They even use their sexual partners’ money to buy sex. There are two kinds of teahouses in County A: pure teahouses for daily life interaction which is good for health [40, 41], and teahouses as sexual venues where older-aged FSW sell sex [6] normally at low price without condom use. Visiting FSW is financially and geographically accessible.

Lack of daily intimate relationship is risky for elderly men who visit FSW. This study confirms that close with their sexual partners in daily life, and being satisfied with their sexual life help elderly men who visit FSW avoid being HIV infected [13, 23, 25–30], but neither cases nor controls got enough social support from their most intimate sexual partners. Their needs of social support especially emotional support are ignored [21, 22]. This study shows that elderly men’s sexual desire declines after the age of 50 as a natural phenomenon [16]. Due to this reason [23], as a common life for elderly people in China, grand-parenting often results in separating for elderly couples [45]. Being separated from their spouse, elderly men not only can’t fulfill their sexual need, but also lack of emotional support. Cases were more dissatisfied with their most intimate sexual partners either in terms of daily life or sexual life than controls. They take an adventure to visit FSW [13, 23, 46] for seeking sexual satisfaction more than “purely fulfil sexual desire” or “loneliness”. They visit different FSW, and become HIV-positive. Instead of condom, intrauterine device (IUD) for women has been main contraception method among married couples. Some elderly men never use condom in their whole life [24]. Low educated elderly men have minimal knowledge about HIV/AIDS [31], and lack of knowledge and skill of condom use result in that the older their first visit to FSW, the riskier they become HIV-positive. There are seven optional reasons for visiting FSW, but only the obscure reason as “other” is a risk factor. The case group was informed about their HIV positive statuses before the interview. The choice of visiting FSW for other reason reflect their regretful attitude [12]. In fact, cases do not concern social norms against visiting FSW [12, 23], their first visit happen mainly within their township rather that migrate sites.

As expected [2], getting HRHE is a protective social interaction but very rare for cases (23.58%). Men use condom inconsistently especially when they feel close with FSW [13]. Interestingly, visiting FSW due to “loneliness” is a protective factor for avoiding HIV infection in this study. We did not measure the frequency of visiting FSW in this study, both cases and controls admitted visiting FSW in their lifetime. Instead of among general elderly men, all discussions are in the context among the ones who visit FSW. The purposes of these elderly men who visit FSW due to “loneliness” are to fulfil their emotional need [13, 23, 25–30]. Through visiting FSW, they succeed to cope with “loneliness” [32, 33], and may visit less frequently than others.

In the future, besides continuously providing HRHE and condom use promotion among elderly men [2, 4], considering them as “receivers” of social support especially emotional support are necessary [39–41]. Involvement of stakeholders such as family members and owners of teahouses are needed. The former plays the role of “givers” of social support to elderly men. The latter are targeting group for outreach of HRHE. Moreover, intensive intervention for rural FSW need to be continuously strengthened.

## Limitations

In the current study, self-report behavior information should be noted. Face-to-face interviews may heighten socially desirable responses such as low report of high-risk sexual behaviors. In order to confront the problems, our interviewers were well trained, interviews were conducted in separate rooms, and local slang was used.

Moreover, in-depth interviews were conducted by skilled medical staff members from County A CDC for case group, but by well-trained young male interviewers for control group. The case group was informed about their HIV positive statuses before the interview. In order to get professional suggestions from the interviewers for their future treatment if they agreed to take part in the study, report bias was at minimal level. Control group knew their HIV negative statuses after the interview. When they admitted visiting FSW in their lifetime, they were open to talk about their sexual experiences to our interviewers. In order to minimize informational bias due to different types of interviewers between two groups, interviewers were trained by the same trainers, only well-trained interviewers conducted the in-depth interview, and interviewers followed the same procedure.

## Conclusions

Cases visit more FSW than controls. The majority of cases never use condom when visiting FSW. Elderly men’s daily life interactions are mainly visiting teahouse which is a potential sexual venue with friends and playing mahjong. Visiting different FSW and for obscure reasons are risky for becoming HIV-positive. The older they start to visit FSW, the more possibility becoming HIV-positive is. Getting HRHE is a formal protective social interaction but very rare for cases (23.58%). Living with spouses is protective, but more than 40% cases do not do so. Social support from the most intimate sexual partner is not enough. Emotional support is protective meanwhile material support only is risky for becoming HIV-positive.

## Data Availability

The datasets analyzed during the current study are available from the corresponding authors on reasonable request.
